# Comparing GABA-dependent physiological measures of inhibition with proton magnetic resonance spectroscopy measurement of GABA using ultra-high-field MRI

**DOI:** 10.1016/j.neuroimage.2017.03.011

**Published:** 2017-05-15

**Authors:** Katherine Dyke, Sophia E. Pépés, Chen Chen, Soyoung Kim, Hilmar P. Sigurdsson, Amelia Draper, Masud Husain, Parashkev Nachev, Penelope A. Gowland, Peter G. Morris, Stephen R. Jackson

**Affiliations:** a School of Psychology, University of Nottingham, UK; b Sir Peter Mansfield Imaging Centre, University of Nottingham, UK; c Institute of Mental Health, School of Medicine, University of Nottingham, UK; d Nuffield Department of Clinical Neuroscience, University of Oxford, UK; e Institute of Cognitive Neuroscience, University College London, UK

## Abstract

Imbalances in glutamatergic (excitatory) and GABA (inhibitory) signalling within key brain networks are thought to underlie many brain and mental health disorders, and for this reason there is considerable interest in investigating how individual variability in localised concentrations of these molecules relate to brain disorders. Magnetic resonance spectroscopy (MRS) provides a reliable means of measuring, in vivo, concentrations of neurometabolites such as GABA, glutamate and glutamine that can be correlated with brain function and dysfunction. However, an issue of much debate is whether the GABA observed and measured using MRS represents the entire pool of GABA available for measurement (i.e., metabolic, intracellular, and extracellular) or is instead limited to only some portion of it. GABA function can also be investigated indirectly in humans through the use of non-invasive transcranial magnetic stimulation (TMS) techniques that can be used to measure cortical excitability and GABA-mediated physiological inhibition. To investigate this issue further we collected in a single session both types of measurement, i.e., TMS measures of cortical excitability and physiological inhibition and ultra-high-field (7 T) MRS measures of GABA, glutamate and glutamine, from the left sensorimotor cortex of the same group of right-handed individuals. We found that TMS and MRS measures were largely uncorrelated with one another, save for the plateau of the TMS IO curve that was negatively correlated with MRS-Glutamate (Glu) and intra-cortical facilitation (10ms ISI) that was positively associated with MRS-Glutamate concentration. These findings are consistent with the view that the GABA concentrations measured using the MRS largely represent pools of GABA that are linked to tonic rather than phasic inhibition and thus contribute to the inhibitory tone of a brain area rather than GABAergic synaptic transmission.

## Highlights

• ultra-high-field (7 Tesla) MRS used to measure of GABA concentration.
• TMS used to measure cortical excitability and GABA-mediated physiological inhibition.
• Both TMS and MRS measures obtained from primary motor cortex in the same individuals.
• TMS measures of physiological inhibition uncorrelated with MRS measures of GABA.
• MRS may represent pools of GABA linked to tonic rather than phasic inhibition.

## Introduction

GABA is the main inhibitory neurotransmitter in the brain. It is present in 25–50% of synapses and has a critical role in regulating excitability throughout the brain. Dysfunction in GABA signalling is also core to many common neurological and psychiatric conditions: including neurodevelopmental disorders such as autism and Tourette syndrome (Clarke et al., 2012, Ramamoorthi and Lin, 2011). Consistent with this view, post-mortem investigations of Tourette syndrome (TS) have demonstrated that there are substantial *decreases* (~50%) in the number of GABA interneurons within the striatum (Kalanithi et al., 2005), and positron emission tomography (PET) imaging studies have reported widespread alterations in GABA_A_ receptor binding throughout the brain in TS, including in particular the striatum, thalamus and insula cortex (Lerner et al., 2012). Most importantly, with respect to the focus of the current study, transcranial magnetic stimulation (TMS) studies of physiological inhibition have repeatedly demonstrated reduced short interval intra-cortical inhibition (SICI) in TS (Gilbert et al., 2004, Heise et al., 2010, Orth et al., 2008). As SICI is thought to be predominantly dependent on GABA-A receptor activity (see Ziemann (2013) for a review) these findings have suggested a role of dysfunctional GABAergic signalling in TS.

Non-invasive *in-vivo* investigation of localised concentrations of GABA within the brain is possible using proton magnetic resonance spectroscopy (MRS). Detection of some neurometabolites, such 1H MRS can be challenging due to low concentration and signal overlap with more concentrated metabolites. Ultra-high field offers the advantages of increased spectral resolution and signal-to-noise ratio (SNR) compared to lower field strengths (Choi et al., 2010, Stephenson et al., 2011, Tkáč et al., 2001, Tkáč et al., 2009). In the context of the current study it is of interest to note that MRS studies of GABA concentration within the cortical motor areas in TS have reported either no differences in GABA concentration (Draper et al., 2014, Puts et al., 2015, Tinaz et al., 2014) or increased levels of GABA relative to age-matched controls (Draper et al., 2014).

The findings outlined above in relation to TS illustrate an apparent contradiction. Specifically, while numerous TMS studies have reported that GABA-A mediated physiological inhibition (SICI) is substantially reduced in TS (Gilbert et al., 2005, Orth et al., 2005, Ziemann et al., 1997) recent MRS studies has reported either no differences in GABA concentration, or increased GABA concentrations within the SMA (Draper et al., 2014) that are associated with decreased gain in motor cortical excitability Draper et al., 2014), a finding that has been widely reported in TS (Draper and Jackson, 2015, Heise et al., 2010). However, it is important to note that these apparently contradictory results are only problematic if we assume that TMS measurements of GABA-mediated inhibition and MRS measurements of localised GABA concentrations are indexing the same inhibitory function.

It is widely accepted that TMS measures of GABA-mediated physiological inhibition such as SICI are likely to be indexing the operation of low threshold, transiently activated, cortical GABA interneurons (Ziemann et al., 1996, Ziemann et al., 1996). In particular, pharmacological studies suggest that SICI measured using inter stimulus intervals (ISI) of 1.5-5ms are largely dependent on GABA-A receptor activity (Hanajima and Ugawa, 2008, Ziemann, 2013, 2015). By contrast, it has been argued that the GABA observed and measured using MRS largely represents extracellular concentrations of GABA that are linked to the ‘tonic’, GABA-ergic, inhibitory tone of a localised brain region (Rae, 2014, Stagg, 2014). If this is indeed the case, then it would be unsurprising that both decreased transient ‘phasic’ inhibition and increased ‘tonic’ inhibition could be observed within a particular patient group, particularly if the latter can be viewed as the consequence of an adaptive, compensatory, response to the former, as has been argued by several authors (Draper et al., 2014, Heise et al., 2010).

Two recent studies sought to address this issue directly by investigating the relationship between TMS measures of SICI and MRS measures of GABA within the primary sensorimotor cortex [PMC] (Stagg et al., 2011, Tremblay et al., 2013) collected from the same individuals. Both studies acquired MRS data at conventional MR field strengths (3 T), and one study investigated GABA concentrations using a GABA-edited (MEGA-PRESS) sequence (Tremblay et al., 2013). The results of these studies are inconclusive. Stagg et al. (2011) reported that MRS-GABA concentrations within the PMC were uncorrelated with 2.5ms SICI measurements, but were significantly correlated with the slope of the 1ms SICI curve. The physiological mechanisms underlying 1ms SICI effects are thought to be distinct from those occurring at later ISIs and although these mechanisms are not yet fully understood, it has been suggested that the effects may reflect axonal refractory periods (Fisher et al., 2002). Stagg et al. (2011) suggest that MRS measures extra-synaptic, tonic inhibition and that GABA that may be associated with the duration of the refractory periods of neuronal axons.

Tremblay et al. (2013) also reported that MRS-GABA concentrations within the PMC were uncorrelated with any TMS measurements, including 3ms SICI, but these authors did not measure 1ms SICI, so we cannot know if the 1ms SICI effect observed by Stagg et al. (2011) is replicable.

The Stagg and Tremblay studies also differ in the associations that they reported for other TMS/MRS metabolite pairings, partially due to the use of different TMS measurements. Thus, while Stagg et al. (2011) reported that a general measure of cortical excitability, the slope of the TMS recruitment (IO) curve, was significantly associated with MRS glutamate concentrations within the PMC, Tremblay et al. (2013) reported a significant positive correlation between Glx (a composite measure of glutamate and glutamine) and the duration of the cortical silent period (CSP).

Furthermore, in addition to differing on which particular TMS measures to relate to MRS measures of motor cortical excitability and physiological inhibition, the above studies also differed in that one study used a GABA-edited MRS sequence (Tremblay et al., 2013) while the other (Stagg et al., 2011) did not. This may have had important implications for the measurements obtained, particularly as both studies were conducted at conventional MR field strengths.

The advantages of ultra-high field (7 T) are the increased signal-to-noise ratio (SNR) obtained and greater chemical shift dispersion (Tkáč et al., 2009). The increased SNR improves the detection sensitivity and efficiency of metabolites, especially those with low concentration such as GABA. Greater chemical shift dispersion increases the separation of signals with similar resonance frequencies, allowing a more accurate identification and quantification of each metabolite. For instance, due to spectral overlapping the differentiation of GABA, Glu and Gln signals are difficult in ^1^H spectra at field strengths of 3 T or less (Puts and Edden, 2012), and Glx (a composite measure of Glu + Gln) is reported instead. By contrast, GABA, Glu and Gln become separable at field strengths of 7 T or above. Also, GABA-edited (J-difference editing) sequences (Mescher et al., 1998, Near et al., 2013) rely on subtraction to remove overlapping signals from the spectrum. This technique is therefore particularly susceptible to motion-related errors that are less of an issue for non-edited MRS sequences (e.g., STEAM) that can be utilised at 7 T (Bhattacharyya et al., 2007, Bogner et al., 2014).

In the current study, we investigated this question by directly comparing a wide range of TMS measurements of motor cortical excitability (including TMS recruitment curves and paired-pulse measures of intra-cortical facilitation [ICF]) and physiological inhibition (including both 1ms and 3ms SICI) with MRS measures of GABA, Glu and Gln acquired at ultra-high-field (7 T) using a non-edited STEAM sequence.

## Subjects

29 healthy right handed adults (age range 19–27) participated in the study. All participants were free from neurological or psychiatric illness and any contraindications for MR scanning or TMS. Of the 29 participants recruited, two were subsequently excluded from the analysis: one due to poor quality TMS data and one due to poor quality MRS data (details below). Details of the 27 participants included in the data analyses are contained in Table 1.

**Table 1:** Participant demographics. Data are presented as mean value ± sd. RMT = mean resting motor threshold. SI 1 mV = mean stimulator output required to produce a MEP with an amplitude of 1 mV. † Percentage of maximal stimulator intensity.

N	Sex (M/F)	Age	rMT †	S1 1MV †
27	13/14	23.1±2.4	45.7 ±6	55.3±6.8

## MR acquisition

All MR data were acquired using an ultra-high field 7 T Philips Achieva system (Philips Healthcare, Best, Netherlands) with a 32-channel radio frequency head coil at the Sir Peter Mansfield Imaging Centre (SPMIC), University of Nottingham. Participants were placed supine and head-first into the scanner. Foam pads were inserted between the participant's head and the coil to minimise and control head movement; a pair of prism glasses were provided to allow participants to view a screen outside the magnet bore.

At the start of each imaging session ^1^H image localiser and B0 maps were acquired, followed by BOLD-fMRI T_2_^*^-weighted images, which were acquired to guide placement of the left primary motor cortex (PMC) spectroscopy voxel in the following MRS scans. The BOLD-fMRI used a single shot EPI sequence (TR/TE=1999/25 ms, FOV=208×192 mm3, matrix=112×112, 30 slices, slice thickness=4mm, no slice gap, 160 dynamics). During the fMRI scan, eight blocks of bimanual finger-to-thumb opposition tapping were performed in a blocked-trial paradigm as follows. The words ‘TAP’ and ‘REST’ were alternately displayed for 8 s and 32 s, respectively. Participants were asked to tap their thumbs to each finger with both hands simultaneously and continuously during the ‘TAP’ phase and to rest (withhold movement) during the ‘REST’ phase. Maximum activation of the left M1 was found by analysing the BOLD response on-line using the Philips IViewBOLD software.

T_1_-weighted anatomical images were then acquired with a MPRAGE sequence (TR/TE/TI=7.3/3.4/998 ms, FA=8^o^, FOV=224×224×120 mm^3^, isotropic resolution=1mm^3^) for tissue segmentation. Anatomical landmarks from these images were also used to assist in the placement of the left PMC voxel for MRS.

**Fig. 1:**
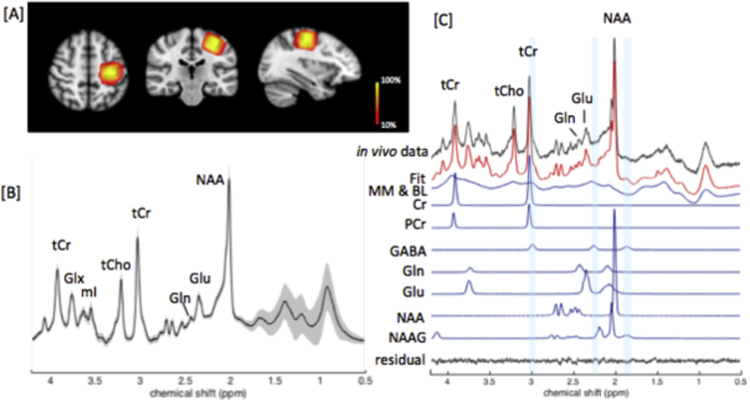
[A] Represents the overlap of the voxel of interest (VOI=20x20×20 mm^3^) over the left hand area of M1 shown in (i) sagittal, (ii) axial (iii) and coronal views. The colour bar represents the percentage MRS voxel overlap across 27 subjects (from 10–100%). The voxel positions from each subject were transformed into a standard brain space (as shown in the Figure) to calculate the percentage of voxel overlap. [B] Standard deviations (shaded area) overlying the group mean in vivo spectrum acquired from the VOI obtained with the STEAM sequence (TE/M=17/17 ms) at 7Tare shown. [C] A representative in vivo spectrum obtained from the M1 VOI is shown, together with its LCModel fit. Residual, and fitted signals for metabolites of interest and macromolecules (MM), baseline (BL) are also shown.

In vivo ^1^H MRS data were acquired from a voxel of interest (VOI=20×20×20mm^3^) placed over the hand area in the left M1 (Fig. 1[A]) using a STEAM sequence (TE/TM/TR=17/17/2000ms, sample size=4096, spectral bandwidth=4000 Hz, phase cycling=8, 288 averages, 9.6 min). Water suppression was performed using multiply optimised insensitive suppression train (MOIST) (Murdoch, 1993). Prior to this, a non-suppressed water reference spectrum (16 averages) from the same VOI was acquired for eddy current correction and quantification. B0 shimming of the VOI was performed automatically by the Philips pencil beam (PB) algorithm (Gruetter, 1993) in order to increase B0 field homogeneity.

## Test – retest reliability and reproducibility of MRS measurements

To determine the reliability and consistency of our MRS measurements we examined the test-retest reliability of our MRS data. Specifically, an additional dataset was obtained from a sub-set of 12 participants who were selected randomly from the sample described above. This second MRS dataset was collected on the same day, approximately 3 h after, the initial MRS dataset was collected. It should be noted that both datasets were collected in an identical manner, and followed the identical procedure as outlined above.

Intra-class correlation coefficient (ICC (2,1)) analyses were used to explore the reliability of key MRS measures (i.e., GABA/tCr, Glu/tCr, Gln/tCr,) across the two sessions. ICC results are reported based upon Lahey et al. (1983), whereby ICC values of <0.4 are considered to indicate poor intra-class reliability, values >0.4 and <0.59 indicate fair intra-class reliability, values >0.6 and <0.74 indicate good intra-class reliability, and values >0.74 indicate excellent intra-class reliability. Negative ICC values are taken to indicate a lack of reliability within the measure.

The ICC(2,1) analysis of GABA/tCr yielded an intra-class correlation coefficient of 0.62 which indicates good intra-class reliability. The ICC(2,1) analysis of Glu/tCr yielded an intra-class correlation coefficient of 0.61 which also indicates good intra-class reliability. The ICC(2,1) analysis of Gln/tCr yielded an intra-class correlation coefficient of 0.44 which indicates fair intra-class reliability.

A previous study by Wijtenburg and colleagues (Wijtenburg et al., 2013) reported on the reproducibility of MRS GABA measurements and found that MRS GABA concentrations varied across brain regions. Specifically, they reported reproducibility (indexed by the coefficient of variation [CV]) was different in the anterior cingulate cortex (CV = 3.2%) and the dorsolateral prefrontal cortex (CV = 16.2%). As this study did not investigate the reproducibility of GABA within the primary motor cortex we report these measurements here. We calculated the CV for each individual across the two testing sessions as outlined above. The mean and median values for the group were as follows: GABA mean CV = 11.91% (median = 5.56%); Glu mean CV = 3.52% (median = 3.46%); Gln mean CV = 18.05% (median = 12.66%). These data confirm that GABA reproducibility in the primary motor cortex are comparable to the data reported for other brain areas by Wijtenburg et al. (2013).

## TMS measurements and EMG recording

MR scanning sessions were performed before TMS measurements were obtained. On average the time in between the final MR scan and the commencement of the collection of TMS measurements was 30 min.

TMS was delivered using a Magstim Bistim system (Magstim, Whiteland, Dyfed, UK) with a figure-of-eight magnetic coil (70mm diameter of each winding). The coil was held tangentially to the scalp and positioned 45° from the midline, resulting in a posterior to anterior current flow. Neuronavigation software (Brain site, Rogue research Inc., Montreal Quebec, Canada) was used in conjunction with individual T_1_-weighted anatomical images (acquired using the MPRAGE sequence outlined above) to aid coil placement over the hand area of the left primary motor cortex. All TMS measures were obtained from the motor hot spot identified for the First Dorsal Interosseous (FDI) muscle. This was defined as the location that consistently yielded the largest MEP amplitudes for the FDI. Participants were required to remain still during testing, and head movements were minimised with the aid of a chin rest. Participants were offered frequent breaks to stretch and adjust their position and neuronavigation was used to accurately reposition the coil over the motor hot spot on each occasion. The coil was held stable over the hot spot using a Manfrotto mechanical arm (Vitec Group, Italy) and adjusted when necessary.

Motor evoked potentials (MEPs) were recorded using disposable Ag-AgCl surface electrodes attached to the right FDI muscle in a belly-tendon montage. The signals were amplified and bandpass filtered (10 Hz–2 kHz, sampling rate 5 kHz) then digitalized using Brainamp ExG (Brain Products GmbH, Gilching, Germany) controlled by Brain Vision Recorder (Brain Products GmbH, Gilching, Germany). Participants were encouraged to maintain their hand in a relaxed position throughout testing.

All trials were controlled using an in-house program (written using Matlab: Mathworks, MA, USA), with an inter-trial interval of 5 s occurring between each trial for all measures. Intra-cortical inhibition and facilitation were investigated using a range of TMS paired pulse protocols, with a range of inter-stimulus intervals (ISIs) including 1, 3, 10 and 12 ms (Kujirai et al., 1993). A 100 ms ISI inhibitory protocol was also measured (Claus et al., 1992, Valls-Sole et al., 1992). All paired pulse measures and unconditioned trials were randomized and presented within the same session. Input output curves were always calculated prior to paired pulse measures.

### Threshold determination

Resting motor threshold (RMT) was determined as the lowest intensity needed to yield an MEP with a peak-to-peak amplitude of >50 µV in the relaxed FDI muscle in a minimum of 5 of 10 trials. A 1 mV (SI 1 mV) threshold was also determined by calculating the lowest intensity needed to evoke an MEP of 1 mV in 5 of 10 consecutive trials.

### Input output curves

TMS intensities at 100, 110, 120, 130, 140 and 150% of RMT were used. 10 pulses at each of the 6 intensities were delivered in a randomized order.

### Unconditioned trials

**Fig. 2:**
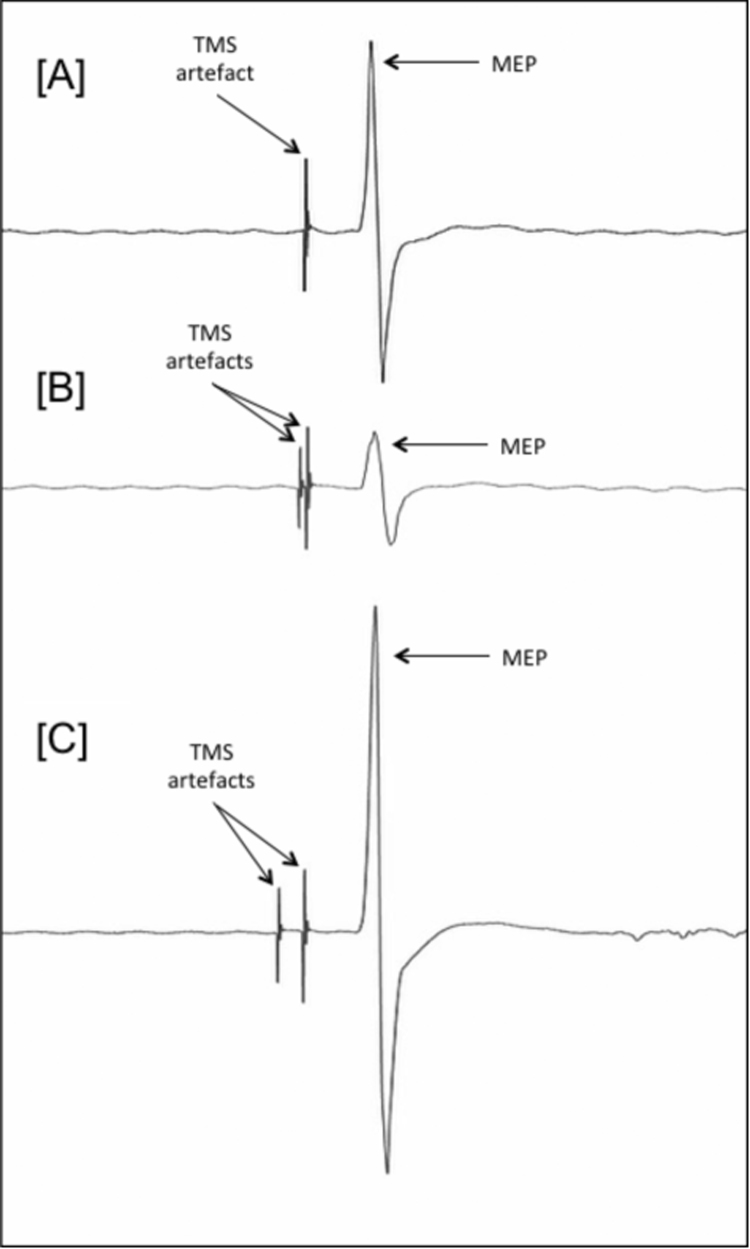
Illustrates representative MEPs. [A] shows an MEP from an unconditioned trial following the delivery of a single TMS test stimulus (SI 1 mv); [B] shows an MEP for a paired-pulse SICI trial. In this case the same test stimulus that is preceded (3 ms) by a sub-threshold CS; and, [C] shows an MEP for a paired-pulse ICF trial. In this case the test stimulus is preceded (10 ms) by a sub-threshold CS.

A total of 30 unconditioned trials were measured at SI 1 mV (Fig. 2A).

### Short interval intracortical inhibition (SICI)

SICI was measured using 1 and 3 ms ISIs. The selection of conditioning stimuli (CS) intensities was informed by a pilot study (data not shown) which revealed 1 ms SICI to have a lower threshold than that of 3ms SICI. This finding confirms previous research (Fisher et al., 2002) and therefore CS intensities of 45%, 50%, 55% and 60% RMT were used to measure 1ms SICI, whereas 60%, 65%, 70% and 75% RMT were used to measure 3ms SICI. Each CS was followed by a suprathreshold test stimulus (TS) of SI 1 mV delivered to the same location (Fig. 2B). Ten trials were measured for each CS-TS pairing for both 1 and 3 ms ISIs.

### Long interval intra-cortical inhibition (LICI)

A single ISI of 100 ms was tested using a suprathreshold CS of 110% RMT and a TS delivered at SI 1 mV. A total of 20 trials were measured.

### Intracortical facilitation (ICF)

10 and 12 ms ISIs were measured using a CS at 75% RMT followed by a SI 1 mV TS (Fig. 2C). 20 trials were measured for each ISI.

## Results

### Analyses of MRS data

*In vivo* ^1^H spectra were fitted and quantified with the LCModel software package (Provencher, 1993). The basis set used for quantification included an experimentally acquired macromolecule spectrum and model spectra of 20 metabolites. The LCModel analysis was performed within the chemical shift range 0.5 to 4.2 ppm. Water scaling was applied using the non-suppressed water reference. The LCmodel control parameters were based on previously published parameters (Tkáč et al., 2009). The absolute concentration for each cerebral metabolite is reported in institutional units. Metabolites with Cramer-Rao lower bound (CRLB) >30% were rejected from further analysis. Mean ± SD CRLB for Glu was 2% ± 0%, for Gln this was 6.98% ± 2.76% and for GABA this was 9.9% ± 4.6%. The line width of in vivo spectra were 10.35±1.99 Hz. Total Cr (tCr, i.e., PCr+Cr) was used as the internal reference for quantification due to its relatively high and stable concentration in the human brain (Danielsen and Ross, 1999, Stagg and Rothman, 2014).The group mean in vivo spectrum acquired from the VOI is presented in Fig. 1[B]. Any significant outliers were identified and removed using Grubbs test.

### Analyses of TMS data

All trials were carefully inspected visually and trials in which there was evidence of pre-contraction of the FDI muscle in the period 500ms prior to an MEP were excluded. For the remaining data, peak-to-peak MEP amplitudes were measured using in house software (programmed using Matlab, Mathworks, MA, USA). When analysing individual participant data, median values were calculated to indicate average MEP amplitude in response to a particular stimulator output. The mean of median values was calculated across the participants for each dependent variable. Any significant outliers in group mean values were identified and removed prior to analyses using Grubbs test.

#### Resting motor thresholds (RMT)

The group mean RMT was 45.7 (±6) of maximum stimulator output. The group mean for the 1mV (SI mV) threshold (i.e., the lowest intensity needed to evoke an MEP of 1mV in 5 of 10 consecutive trials) was 55.3(±6.8) %. The group mean MEP amplitude for a TMS pulse delivered at SI 1mV was 1365.3 (±556.9)mV.

#### TMS recruitment (IO) curves

Single pulse TMS IO curves were measured by calculating for each individual the median MEP amplitude for each given TMS intensity (100−150% RMT). Four-parameter sigmoidal fits were then applied to the resultant values and the maximal slope and the plateau of the curve were calculated. The sigmoidal function used to fit curves to the individual datasets was:

$${\mathrm{MEP}}_{S}\;=\;y_{0}\;+\;\frac{{\mathrm{MEP}}_{\mathrm{MAX}}}{1+10^{(S_{50}-S)k}}$$

MEP_MAX_ is the maximum MEP amplitude measured, S_50_ is the TMS intensity needed to produce 50% of the maximum MEP, k is the gradient of the maximum steepness of the curve and y_0_ is the minimal MEP response, which was set to 0.

**Fig. 3:**
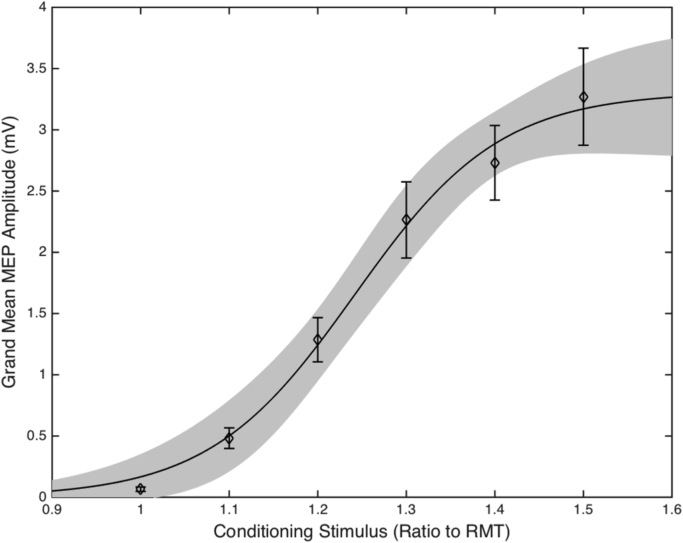
Group mean of individual median MEPs following stimulation of 100–150% of individual RMT. The errors bars are SEM and the shaded region is the 95% confidence intervals of the sigmoidal function fitted to the data.

Group IO curve data are presented in Fig. 3. Inspection of this figure clearly illustrates that in our study, increased TMS stimulator intensity led to increased MEP amplitudes. This was confirmed by statistical analysis using a one way ANOVA which yielded a significant effect of stimulator intensity (F[5,156] = 26.08, p < 0.0001). Two measures of interest were taken from the sigmoid curve: the maximum slope of the sigmoid (IO curve slope) and plateau of the sigmoid (IO plateau).

#### Paired pulse data

**Fig. 4:**
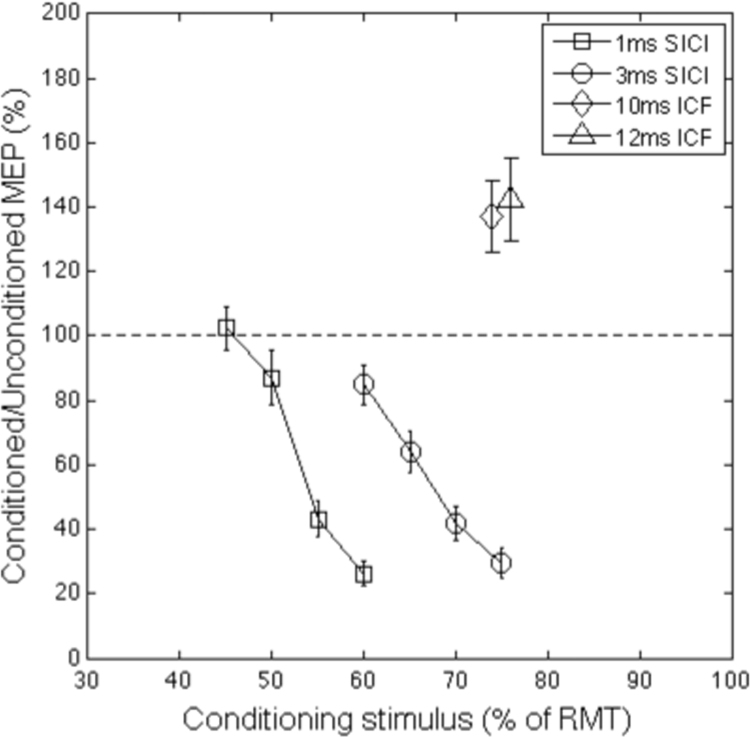
Group mean of individual median MEP values for paired pulse SICI (1 ms and 3 ms ISI) and ICF (10 ms and 12 ms ISI) trials. Error bars represent the standard error of the mean.

Paired pulse trials were analysed by calculating for each individual the median MEP amplitude for each CS intensity at each ISI. These values were then divided by the median MEP amplitude for unconditioned trials to create a ratio measure. Linear slopes were fitted to 1ms and 3ms SICI measures (Fig. 4) and a median value was calculated across the different CS intensities to reveal the average level of inhibition. Individual median MEP values were also calculated for ICF trials, the resultant group data are presented in Fig. 4. Inspection of this figure clearly illustrates that while the SICI paired pulse trials (ISIs 1 ms and 3 ms) led to a large reduction of the MEP produced by the test stimulus, the ICF trials (ISIs 10 ms and 12 ms) led to a large increase in the MEP to the test stimulus. These data are consistent with the inhibitory and excitatory effects of SICI and ICF paired pulse TMS stimulation. Statistical analysis using a one way ANOVA confirmed that there was a significant effect of stimulator intensity on MEP amplitudes for both the 1 ms SICI curve (F(3,104)=32.58, p<0.0001) and the 3 ms SICI curve (F(3,107) = 19.23, p < 0.0001). Paired sample t-tests confirmed that the values for both 10 ms ICF trials (*t*(26)=10.8, *p*=.000) and 12 ms ICF (*t*(26)=10.63, *p*=.0001) differed significantly from unconditioned trials.

Following initial analyses, it was determined that data from LICI trials would not be analysed further. This was based upon the large percentage of participants who exhibited floor levels for this measure (i.e., there was a complete inhibition of the MEP in 8/27 participants). While this illustrates that the LICI paired pulse TMS procedure was highly effective, it reduces individual variability in LICI values and therefore makes the correlation analyses less effective.

## Correlations between MRS and TMS measures

The key focus of this study was to directly examine the association between TMS measures of physiological inhibition - assessed by TMS stimulation of the hand area of the primary motor cortex (PMC) of the left hemisphere - with MRS measurement of GABA concentration – acquired from a voxel centred over the hand area of the PMC of the left hemisphere. To examine this association Pearson correlation coefficients were calculated for the entire set of TMS measures against a measure of PMC GABA concentration. For completeness correlations are reported without adjustment for multiple comparisons. Where correlation coefficients are statistically significant we report the adjusted statistical threshold (alpha) calculated for multiple comparisons using the Holm-Bonferroni correction method for each neurotransmitter separately.

The TMS measures used in the analyses consisted of the following ten measurements for each individual: the resting motor threshold (**RMT 50–100 μV**); the median MEP for a single, unconditioned, TMS stimulus delivered at that individual's RMT (**med MEP**); the stimulator output (%) required to produce an MEP of approximately 1 mV (**SI 1 mV**); the linear slope of the TMS IO curve (**IO curve**); the median inhibition value (%) observed for 1ms SICI trials (**1 ms SICI**); the linear slope value for 1 ms SICI trials (**1 ms SICI slope**); the median inhibition value (%) observed for 3 ms SICI trials (**3 ms SICI**); the linear slope value for 3 ms SICI trials (**3 ms SICI slope**); the median value (%) observed for 10ms ICF trials (**10 ms ICF**); and, the median value (%) observed for 12ms ICF trials (**12 ms ICF**).

We used the ratio of GABA to total Creatine (tCr) as our measure of GABA concentration. For completeness, and to maintain comparability with previous studies including our own, we also investigated the ratio of GABA to *N*-acetylaspartate (NAA). These analyses yielded similar results to the tCr ratio data and in the interests of brevity are not reported here. In addition, while the primary focus of this study was to examine the association between TMS measures of physiological inhibition (e.g., SICI) and MRS measures of GABA (hereafter referred to as MRS-GABA), for completeness we report the relationship of our TMS measures with MRS measures of Glutamate (Glu), Glutamine (Gln), and the ratio of Glutamine to Glutamate.

### Association of TMS measures with GABA

#### GABA/tCr

**Fig. 5:**
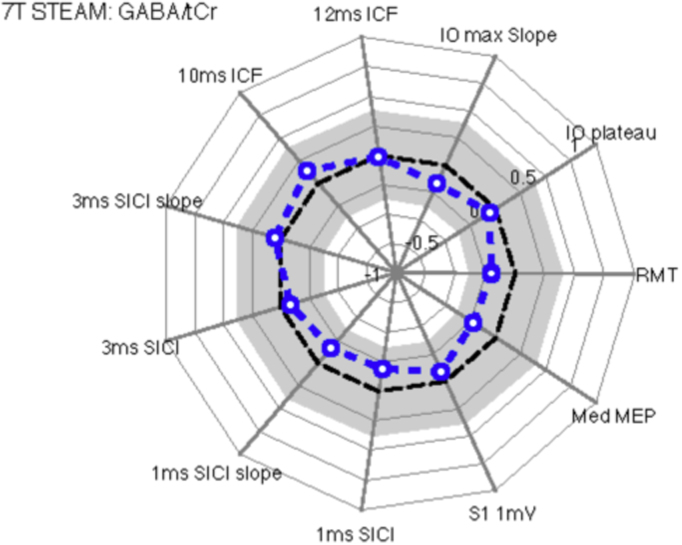
Spider's web plots illustrate Pearson correlation coefficients for individual values for each TMS measurement relative to specific MRS measures (metabolite ratios). This figure illustrates Pearson correlation coefficients for TMS measures with GABA/tCr ratios. The plot presents correlation coefficients running from 1.0 (outer ring) to −1.0 (inner ring), with the broken black line representing a correlation coefficient of zero. The grey region represents non-significant correlation coefficient values. The broken blue line represents the observed correlation coefficients for each TMS measure. Open blue circles are not statistically significant (p >= 0.05) and filled blue circles represent statistically significant correlations (p < 0.05). The analyses revealed that there were no statistically significant correlations between GABA concentration and any TMS measure.

Fig. 5 displays a spider plot of the Pearson correlation coefficients of the TMS measures with GABA/tCr ratio. These analyses revealed that all effects failed to reach conventional levels of statistical significance (all *p* > 0.29). In particular, the correlation coefficients for 1 ms and 3 ms SICI did not approach statistical significance for either median inhibition (1ms SICI: *r* = −0.19, *p*=0.34; 3 ms SICI: *r* = −0.08, *p*=0.68), or slope (1 ms SICI: *r* = −0.17, *p* = 0.39; 3 ms SICI: *r* = 0.06, *p* = 0.77).

### Association of TMS measures with glutamate

#### Glu/tCr

**Fig. 6:**
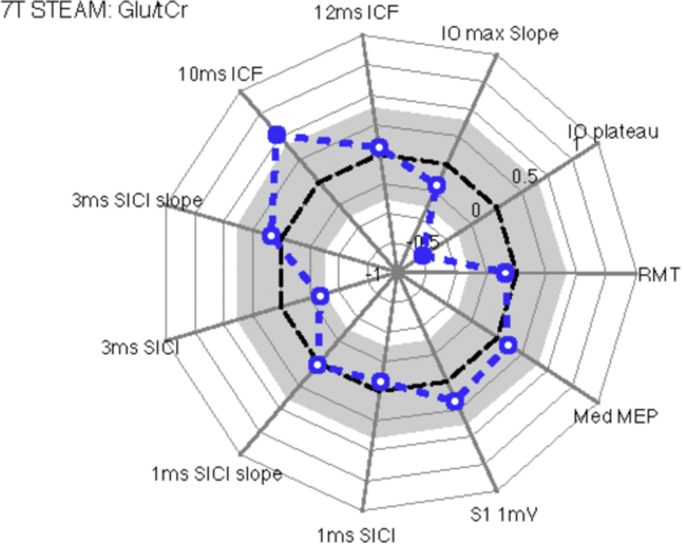
Spider web plots that illustrate Pearson correlation coefficients between individual values for each TMS measurement and Glutamate concentrations (Glu/tCr ratios). The analyses revealed that there was a significant positive correlation between glutamate concentration and 10 ms ICF and a negative correlation with the plateau of TMS IO curves.

Fig. 6 displays a spider's web plot of the Pearson correlation coefficients of the TMS measures with Glu/tCr ratio. There was a significant negative relationship between IO plateau and Glu/tCr (*r*=−0.74, *p*<0.0001 [corrected using Holm-Bonferroni method]) but not for IO maximal slope (*r*=−0.197, *p*=0.34) or RMT (*r*=−0.09, *p*=0.67). A significant positive relationship was found between Glu/tCR and 10 ms ICF (ICF 10 ms: r=0.52, p<0.01), however this did not survive Holm-Bonferroni correction (*r*=0.52, *p*=0.08). 12 ms ICF was not found to have any relationship to Glu/tCR (ICF 12 ms: *r*=0.06, *p*=0.78).

### Association of TMS measures with glutamine

#### Gln/tCr

**Fig. 7:**
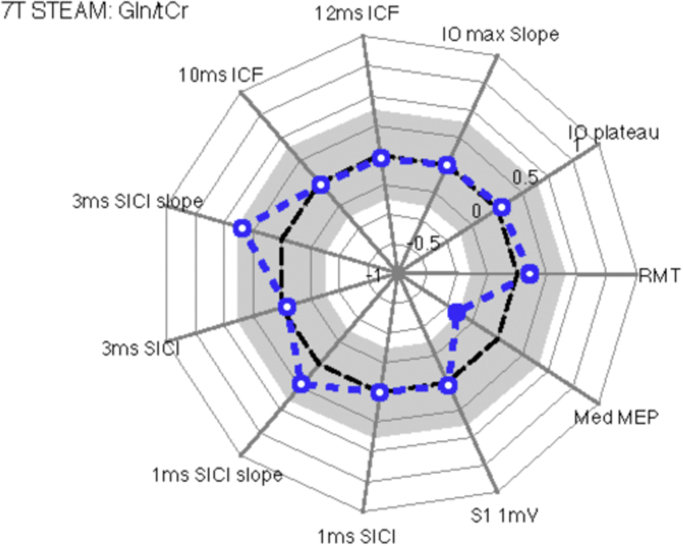
Spider web plots that illustrate Pearson correlation coefficients between individual values for each TMS measurement and Glutamine concentrations (Gln/tCr ratios). The analyses revealed that there was a statistically significant correlation between Gln concentration and the median amplitude of each individual's MEP.

**Fig. 8:**
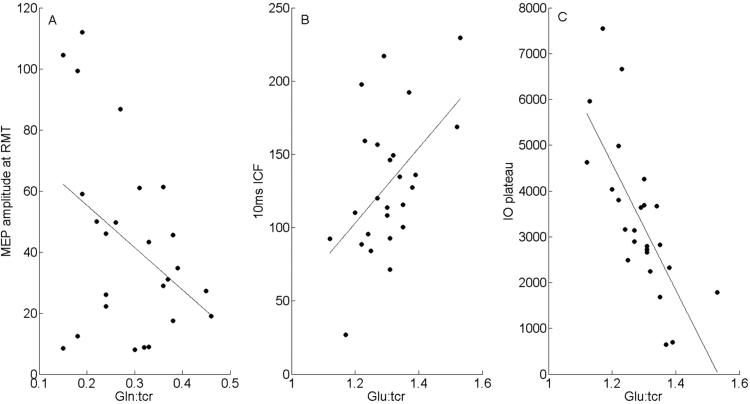
Scatter plots that illustrate the association between TMS measures (Y axis) and MRS measures (X axis) for statistically significant Pearson correlations. A. Illustrates the negative correlation between MEP amplitude and Gln. B. Illustrates the positive correlation between 10 ms ICF and Glu. C. Illustrates the negative correlation between the plateau of the IO curve for each individual and Glu concentration.

Fig. 7 displays a spider's web plot of the Pearson correlation coefficients of the TMS measures with Gln/tCr ratio. The analysis revealed a significant correlation between the Gln/tCr ratio and the median amplitude of MEPs in response to stimulation at RMT (*r*=−0.40, *p*=0.05), although this did not survive the Holm-Bonferroni correction (*r*=−0.4, *p*=0.5). Neither RMT (*r*=0.11, *p*=0.61) nor IO maximal slope (*r*=−0.01, *p*=0.97) were significantly associated with Gln/tCR concentration and all other correlations failed to reach statistical significance. Fig. 8.

### Multiple regression analyses

To address any potential multivariate effects, we entered the following six MRS measures as separate predictors into a multiple regression model: GABA/tCR; Glu/tCR; Gln/tCR; GABA/Glu; GABA/Gln; and Gln/Glu. These predictors were then used to predict each of the TMS measurements in turn.

These analyses confirmed that the magnitude of the median MEP obtained at RMT was predicted by a combination of the Gln/Glu ratio (*t*=−3.428, *p*=0.03) and GABA/Glu ratio (*t*=−2.848, *p*=0.01)(*t* = −3.09, *p* = 0.005); R^2^ = 0.4, F = 7.09566, *p* = 0.004.

The model also revealed that 10ms ICF was predicted by Glu/tCR ratio (*t*=−2.915, *p*=0.008), R^2^= 0.270, F=8.495, *p*=0.008. In addition to this Glu/tCR was found to significantly predict IO plateau (*t*=−2.915, p=.008), R^2^= 0.270, F=8.495, *p*=0.008.

### Bayesian statistics

To overcome potential limitations of conventional correlational analyses, a Bayesian Hypothesis test was conducted. This was used primarily to evaluate whether there was evidence in favour of the null hypothesis (H_0_), i.e. that a metabolite bore no relationship with neurotransmitter function as assessed by TMS. It further allowed us to quantify the strength of the relationship of any correlations found (evidence in support of the alternative hypothesis, H_1_). Bayes Factors (BF_10_) were calculated using JASP (JASP-Team, 2016), JASP uses the Bayesian correlation test proposed by Jeffreys (1961). Bayes Factors above 1 show support for H_1_ whilst below 1 show support for the H_0_, the magnitude of the BF_10_ shows the strength of the evidence in support of either the H_1_ or H_0_. For cut-off values for the different strengths of evidence please see Table 2.

**Table 2:** Category values for BF_10_, table adapted from Wetzels and Wagenmakers (2012).

Bayes factor BF_10_			Interpretation
> 100	–	∞	Decisive evidence for H_1_
> 30	–	< 100	Very strong evidence for H_1_
> 10	–	< 30	Strong evidence for H_1_
> 3	–	< 10	Substantial evidence for H_1_
> 1	–	< 3	Anecdotal evidence for H_1_
	1		No evidence
			
> 1/3	–	< 1	Anecdotal evidence for H_0_
> 1/10	–	< 1/3	Substantial evidence for H_0_
> 1/30	–	< 1/10	Strong evidence for H_0_
> 1/100	–	< 1/30	Very Strong evidence for H_0_
0	–	< 1/100	Decisive evidence for H_0_

In support of previous analysis, the Bayesian Hypothesis test showed there was decisive evidence that Glu/tCr is related to IO plateau (BF_10_=1119.593) and substantial evidence that ICF 10ms is related to Glu/tCr (BF_10_=7.054). Further, there is substantial evidence that the majority of TMS measures do not relate to MRS measures of Glu/tCr, Gln/tCr or GABA/tCr. Most notably there is substantial evidence for no relationship between SICI 3ms and SICI 3ms slope and GABA/tCr (BF_01_=3.857 and 4.017 respectively). For full results of the Bayesian Hypothesis Test please refer to Table 3.

**Table 3:** Bayesian hypothesis te st for correlations. Data presented are each BF_10_ calculated to test the relationship between MRS metabolites and TMS measures.

	**Glu/Cr**	**Gln/Cr**	**GABA/Cr**
RMT	0.276†	0.280†	0.372
MedMEP	0.284†	1.670	0.427
S1 1mV	0.350	0.240†	0.262†
SICI 1ms	0.262†	0.239†	0.366
SICI 1ms Slope	0.244†	0.424	0.339
SICI 3ms	0.881	0.243†	0.259†
SICI 3ms Slope	0.266†	1.020	0.249†
ICF 10ms	7.054*	0.245	0.296†
ICF 12ms	0.253†	0.241†	0.239†
IO Maximal Slope	0.378	0.244†	0.346
IO Plateau	1119.593***	0.246†	0.252†

*Substantial evidence for H1, **strong evidence for H1, ***decisive evidence for H1, †substantial evidence for H0.

BF_10_ > 1 supports H1, BF_10_<1 supports H0, BF_10_=1 suggests no evidence.

## Discussion

Imbalances in glutamatergic (excitatory) and GABA (inhibitory) signalling within key brain networks are thought to underlie many brain disorders, including: schizophrenia; depression; chronic pain; and ‘hyperkinetic’ neurodevelopmental disorders such as TS (Clarke et al., 2012, Ramamoorthi and Lin, 2011) and for this reason there is considerable current interest in the use of MRS to measure *in vivo* concentrations of brain molecules (e.g. GABA, glutamate, glutamine) that can be correlated with brain function and dysfunction. Importantly, while it is tempting to equate the GABA measured using MRS (hereafter referred to as MRS-GABA) with neurotransmitter function, and with physiological or behavioural inhibition (e.g. Dharmadhikari et al. (2015); Haag et al. (2015)) it is important to note that at any point in time only a fraction of MRS-GABA will be neurotransmitter, and that increased MRS-GABA concentrations do not necessarily mean that there is increased physiological or behavioural inhibition (Rae, 2014). Furthermore, it is currently unclear whether MRS-GABA represents the entire pool of GABA available for measurement (i.e., metabolic, intracellular, and extracellular GABA), or as some have argued, represents instead largely extracellular, extra-synaptic, GABA that is unrelated to the synaptic transmission of GABA (Rae, 2014, Stagg, 2014).

In the current study we compared directly, in the same individuals, MRS-GABA (and other important neurometabolites such as glutamate and glutamine) measured from a voxel located in the sensorimotor cortex against TMS measures of cortical excitability and GABA-mediated physiological inhibition measured from the hand area of primary motor cortex. It is important to note that using these techniques we obtained high-quality MRS data and replicated each of the previously reported TMS effects (i.e., TMS recruitment curves, 1 ms SICI and 3 ms SICI curves, and 10 ms and 12 ms ICF effects) that we set out to examine in this study.

The main results of our study are summarised below.

1. Individual concentrations of MRS-GABA were *unrelated* to any TMS measurements, including TMS measures of: general cortical excitability (i.e., TMS recruitment curve slopes); GABA-mediated physiological inhibition (i.e., 1 ms SICI and 3 ms SICI), and TMS measures thought to be dependent upon the glutamatergic NMDA receptor (i.e., 10 ms ICF and 12 ms ICF).
2. Individual levels of MRS-Glutamate (Glu) were significantly negatively correlated with the plateau of the IO curve; a measure which is thought to reflect the balance of excitatory and inhibitory components of the corticospinal volley (Devanne et al., 1997). The relationship suggests that as levels of MRS-Glu increase the maximum MEP amplitude predicted by the model reduces. Multiple regression analysis revealed Glu/tCR to be the only significant predictor of this measure and furthermore Bayesian hypothesis test suggests very strong support in favour of the experimental hypothesis.
3. MRS-Glu was also found to be marginally correlated with 10 ms but not 12 ms ICF. The multiple regression analysis confirmed that Glu/tCR was a significant predictor of 10 ms but not 12 ms ICF. Bayesian hypothesis testing confirmed that there is substantial evidence in favour of the relationship.
4. There was some evidence of correlation between Glutamine (Gln) concentrations and median amplitude of the MEP response to TMS stimulation delivered at RMT. However, the Bayseian hypothesis test revealed only anecdotal support in favour of the relationship. This is possibly explained by the findings of the linear regression model which reveal that median MEP amplitude was found to be predicted by both the Gln/Glu and GABA/Glu ratios, but not significantly by Gln/tCR.

### MRS-GABA and physiological inhibition

Our finding, that individual concentrations of MRS-GABA are unrelated to GABA-mediated physiological inhibition, as measured by 3 ms SICI, replicates previous reports for 3 ms SICI (Tremblay et al., 2013) and 2.5 ms SICI (Stagg et al., 2011). The physiological mechanisms that underpin both 2.5 ms and 3 ms SICI effects are well established and are thought to primarily involve post-synaptic inhibition mediated through GABA-A receptors (Ziemann et al., 2015). Although SICI has also been found to be modulated by neurotransmitters such as dopamine (Gilbert et al., 2006, Korchounov et al., 2007, Ziemann et al., 1996), the contribution that such neurotransmitters make to GABAergic neurotransmission is complex (see (Hasselmo, 1995) and beyond the scope of this article (Hasselmo, 1995). As a result we suggest that the lack of correlation between 3ms SICI and MRS-GABA observed in the current study and also previously indicates that the primary source of MRS-GABA is unlikely to be that associated with GABAergic synaptic transmission, but instead most likely relates to concentrations of metabolic GABA and to levels of ambient extracellular GABA that contribute to tonic GABAergic activity and therefore to the GABAergic tone of a brain region (Rae, 2014, Stagg, 2014).

In a previous study, Stagg and colleagues (Stagg et al., 2011) reported a significant correlation between MRS-GABA concentrations and 1 ms SICI slopes. It is important to note that the underlying physiological mechanisms for the 1 ms SICI effect are thought to be distinct from those associated with longer ISIs such as the 3 ms SICI effect (Cengiz et al., 2013, Fisher et al., 2002, Roshan et al., 2003), however the exact mechanisms underlying 1 ms SICI are currently unclear. Some have proposed that the effects may relate to the refractory periods of inter-neurons (Cengiz et al., 2013, Fisher et al., 2002), whereas others have argued that synaptic processes may also play a role (Roshan et al., 2003, Vucic et al., 2009).

Unfortunately, in the current study we do not replicate the finding observed by Stagg et al. (2011) and we cannot readily account for this. It is unlikely to be any lack of power in the current study as the sample size used here was more that double that used previously (i.e., N=27 versus N=12). Similarly, it is also unlikely to be due to the efficacy of our TMS procedures as our 1ms SICI protocol was highly effective in producing effective inhibition and we clearly observed a 1ms SICI curve. The main differences between the two studies were the nature of the MRS protocol used (i.e. SPECIAL versus STEAM) and the field strength of the MR scanners (i.e., 3 T versus 7 T). Obviously it is difficult to conclude anything from a null effect, so at this point we take the view that the previously observed relationship between 1 ms SICI and MRS-GABA is not entirely reliable.

### MRS-glutamate and glutamine and cortical excitability

TMS recruitment curves are thought to reflect cortical excitability more generally and the strength of cortico-spinal projections (Chen, 2000). However, different physiological processes may contribute to the TMS recruitment curve across the different stimulator intensities used, and various neuromodulators and neurotransmitters, including both GABA and Glu, may contribute to the effects observed (Ziemann, 2013). Furthermore, different aspects of the recruitment curve may relate to different mechanisms. It may be that measures of the slope of the curve are distinct to measures of the plateau for example (Kouchtir-Devanne et al., 2012).

Stagg et al. (2011) reported a significant correlation between cortical excitability, as indexed by the slope of the TMS recruitment curve, and MRS-glutamate. We did not replicate this finding in the current study and in fact found quite the opposite, at least at first appearance. We found the plateau of the IO curve to be negatively correlated with Glu/tCr. We did not, however, find a relationship between IO curve and Glu/tCr. The most obvious differences between both protocols as mentioned are the differences between the sequences used to acquire MRS data, the scanner strength and the sample size. Other possible explanations for the differences between these two studies may be the subtle differences between the protocols used for measuring TMS recruitment curves in each study. In the current study we used a standard procedure; increasing the stimulator intensity in 10% increments proportional to an individual's resting motor threshold. By contrast, Stagg et al. (2011) used a procedure in which the percentage of the intensity needed to yield an MEP of 1mV was tracked over time. This method ensures a similar degree of neuronal recruitment across participants’ mid-slope, whereas the method that we used ensures that this is the case at 100% of RMT. However, it is important to note that despite these procedural differences, the IO curves themselves appear to be similar. In particular, the mean amplitude for MEPs in response to the highest stimulation was highly similar across both experiments (i.e., approximately 3–3.5 mV) indicating that the two methods were producing comparable effects. For this reason we feel that the different results obtained in the two studies are unlikely to have resulted from procedural differences in TMS stimulation.

At first glance our finding appears to be somewhat unorthodox, however, it highlights the importance that what we are measuring with MRS (and TMS) is most probably not just “excitation” vs. “inhibition”. Initially a high plateau may indicate more excitation, in which case, we may expect more glutamate. This is simply not the case. Prior literature describes that the maximum plateau of a recruitment curve does not simply reflect the maximum output of the corticospinal system (Devanne et al., 1997, Houdayer et al., 2008, Kouchtir-Devanne et al., 2012). It may instead reflect the intrinsic balance of excitation and inhibition at the PMC (Devanne et al., 1997). Few articles discuss the cortical origins that lead to the plateau of a recruitment curve. One experiment shows decreases in maximum plateau across different active muscle conditions is accompanied by reductions in SICI and LICI but not in ICF (Kouchtir-Devanne et al., 2012) and have speculated that this may relate to the functional coupling between *intra*cortical circuits within the PMC. At least one study has shown that slope and plateau do not always change together and active high frequency rTMS has been shown to reduce plateau and increase slope (Houdayer et al., 2008), thus our findings may not be entirely incompatible with Stagg's 2011 paper. Any conclusions from this finding can only be speculation on our part, particularly since to our knowledge there are no similar findings in the literature between IO plateau and glutamate (or any other neurometabolite).

A significant relationship was found between 10 ms but not 12 ms ICF and MRS-glutamate. The lack of a significant relationship with 12 ms is consistent with the previous findings by Stagg et al. (2011), however, the relationship with 10 ms ICF is novel as this was not measured in either of the previous TMS-MRS works (Stagg et al., 2011, Tremblay et al., 2013). The mechanisms underlying ICF are not yet fully understood, however, ICF is generally thought to test the excitability of an excitatory neuronal motor network which is likely to be modulated by both glutamatergic and GABAergic mechanisms (Ziemann, 2013). ICF can be measured using ISIs of 7–20 ms (Kujirai et al., 1993, Vucic et al., 2006) and although this is a relatively large range of effective ISIs, to our knowledge no clear distinction has been drawn between these parameters (unlike SICI). Therefore, although the results suggest support for the experimental hypothesis, further study and replication of this effect is warranted to draw strong conclusions about this relationship.

Finally, we demonstrated that individual MEP amplitudes were predicted by a linear combination of the ratios of glutamine/glutamate and GABA/glutamate. Glutamate exists in several metabolic pools in the brain and these pools serve as the source of glutamate for neurotransmission. Also, there is a balanced cycling between glutamate and glutamine that is essential for the normal operation of brain functions and the levels of these neurometabolites are highly correlated with one another in the healthy brain (Rae, 2014). Specifically, Glutamate removed from the synaptic cleft is converted to glutamine within astrocytes and astrocyte-derived glutamine is then used as a precursor for the synthesis of glutamate or GABA within neurons. This cycling of glutamate and glutamine between cell types in the brain is highly dynamic and is thought to account for 80% of cerebral glucose consumption (Ramadan et al., 2013). For this reason it is very likely that TMS-induced changes in cortical excitability may be indexed by subtle changes in the balance between glutamate and glutamine.

Overall the results of the current study reveal mixed support for the previous findings reported thus far in the limited number of studies that have directly compared the relationship between TMS measures of cortical excitability and physiological inhibition (i.e., TMS recruitment curves, ICF and SICI) and key neurometabolites (i.e., glutamate, glutamine and GABA). Specifically, we find that 3 ms SICI and MRS measured GABA are uncorrelated, we interpret these findings as consistent with the view that the GABA concentrations measured using the MRS largely represent pools of extracellular GABA that are linked to tonic rather than phasic inhibition and thus contribute to the inhibitory tone of a brain area rather than GABAergic synaptic transmission (Rae, 2014, Stagg, 2014).

## Conflict of interest statement

We wish to confirm that there are no known conflicts of interest associated with this publication and there has been no significant financial support for this work that could have influenced its outcome.

We confirm that the manuscript has been read and approved by all named authors and that there are no other persons who satisfied the criteria for authorship but are not listed. We further confirm that the order of authors listed in the manuscript has been approved by all of us.

We confirm that we have given due consideration to the protection of intellectual property associated with this work and that there are no impediments to publication, including the timing of publication, with respect to intellectual property. In so doing we confirm that we have followed the regulations of our institutions concerning intellectual property.

We further confirm that any aspect of the work covered in this manuscript that has involved either experimental animals or human patients has been conducted with the ethical approval of all relevant bodies and that such approvals are acknowledged within the manuscript.
